# Low Doses of the Carcinogen Furan Alter Cell Cycle and Apoptosis Gene Expression in Rat Liver Independent of DNA Methylation

**DOI:** 10.1289/ehp.1002153

**Published:** 2010-06-18

**Authors:** Tao Chen, Angela Mally, Sibel Ozden, J. Kevin Chipman

**Affiliations:** 1 School of Biosciences, University of Birmingham, Birmingham, United Kingdom; 2 Department of Toxicology, University of Würzburg, Würzburg, Germany; 3 Department of Pharmaceutical Toxicology, Istanbul University, Istanbul, Turkey

**Keywords:** carcinogenicity, DNA methylation, epigenetic, furan, gene expression, liver, miRNA

## Abstract

**Background:**

Evidence of potent rodent carcinogenicity via an unclear mechanism suggests that furan in various foods [leading to an intake of up to 3.5 μg/kg body weight (bw)/day] may present a potential risk to human health.

**Objectives:**

We tested the hypothesis that altered expression of genes related to cell cycle control, apoptosis, and DNA damage may contribute to the carcinogenicity of furan in rodents. In addition, we investigated the reversibility of such changes and the potential role of epigenetic mechanisms in response to furan doses that approach the maximum estimated dietary intake in humans.

**Methods:**

The mRNA expression profiles of genes related to cell cycle, apoptosis, and DNA damage in rat liver treated with furan concentrations of 0.1 and 2 mg/kg bw were measured by quantitative polymerase chain reaction (PCR) arrays. We assessed epigenetic changes by analysis of global and gene-specific DNA methylation [methylation-specific PCR, combined bisulfite restriction analysis (COBRA), and methylated DNA immunoprecipitation chip] and microRNA (miRNA) analyses.

**Results:**

The expression profiles of apoptosis-related and cell-cycle–related genes were unchanged after 5 days of treatment, although we observed a statistically significant change in the expression of genes related to cell cycle control and apoptosis, but not DNA damage, after 4 weeks of treatment. These changes were reversed after an off-dose period of 2 weeks. None of the gene expression changes was associated with a change in DNA methylation, although we detected minor changes in the miRNA expression profile (5 miRNA alterations out of 349 measured) that may have contributed to modification of gene expression in some cases.

**Conclusion:**

Nongenotoxic changes in gene expression may contribute to the carcinogenicity of furan in rodents. These findings highlight the need for a more comprehensive risk assessment of furan exposure in humans.

Furan is an important industrial compound that was recently found in a number of heated food items, including baby food (Food and Drug Administration 2004). Furan has been reported to cause liver tumors characteristic of hepatocellular carcinoma and cholangiocarcinoma (CC) in both male and female rats at doses of 2, 4, or 8 mg/kg body weight (bw), 5 days a week for 2 years [National Toxicology Program (NTP) 1993]. Male rats treated with 30 mg/kg bw furan for 3 months develop cholangiofibrosis, which in some cases progressed to tumors after 9 or 15 months without further treatment (Maronpot et al. 1991). There are no tumorigenicity data at doses < 2 mg/kg bw. Bearing in mind that CC is the next most common primary hepatic malignancy in humans after hepatocellular carcinoma and that it is associated with high mortality, an assessment of the effects of furan relevant to carcinogenicity at doses < 2 mg/kg is essential. A recent evaluation by the Joint Food and Agriculture Organization/World Health Organization Expert Committee on Food Additives (JECFA) also indicated a human health concern (JECFA 2010).

The mechanisms of furan carcinogenicity are still not well understood. Furan has shown negative results in a number of *in vitro* genotoxicity assays (Mortelmans et al. 1986), and an *in vivo* DNA binding study provided no evidence for covalent binding of furan to DNA (Burka et al. 1991). Wilson et al. (1992) found no evidence of increased DNA synthesis in an *in vivo* DNA repair assay, and furan was not clastogenic or aneugenic in either *in vivo* or *in vitro* studies (Durling et al. 2007). Kellert et al. (2008) demonstrated that furan was not genotoxic when added directly to mouse lymphoma cells, although its metabolite *cis*-2-butene-1,4-dial was genotoxic. Thus, the potential contribution of both genotoxic and nongenotoxic mechanisms to the carcinogenicity of furan must be taken into account.

Carcinogenesis is a complex multistage process during which the tightly controlled balance between cell proliferation and cell death is disrupted. Furan-induced cell proliferation and apoptosis have been found in mouse and rat liver (Fransson-Steen et al. 1997; Mugford et al. 1997; Wilson et al. 1992). Cell proliferation, as a necessary component for tumor development, may influence tumor formation by increasing spontaneous mutations or providing a promotional influence to spontaneously initiated cells. Additionally, secondary oxidative DNA damage has been associated with chronic inflammation after furan exposure (Hickling et al. 2010).

Because of the uncertainty about the relative importance of genetic and epigenetic mechanisms, we investigated potential effects of furan on gene expression in relation to cell cycle, apoptosis, and DNA damage, and we assessed potential changes in DNA methylation and microRNA (miRNA) expression. DNA methylation is a potentially reversible chemical modification of cytosine residues that occurs predominantly in CpG dinucleotides in mammals. Cancer cells often display global hypomethylation, which can cause genomic instability and activation of oncogenes, whereas the promoter region of some specific genes, especially tumor suppressor genes, can be methylated, resulting in transcriptional repression. DNA methylation is somewhat dynamic and responsive to environmental exposures (Szyf 2007). Thus, several nongenotoxic chemicals have been found to affect gene function through changes in DNA methylation (Bombail et al. 2004). For example, the nongenotoxic rodent carcinogen phenobarbital induces global hypomethylation and regional hypermethylation in rodent livers (Bachman et al. 2006; Phillips and Goodman 2009). Aberrant DNA hypermethylation of tumor suppressor genes in human CC has also been frequently reported (Sandhu et al. 2008; Yang et al. 2005). miRNAs are a group of regulatory RNAs of 19–22 nucleotides involved in posttranscriptional gene regulation (Bartel 2004). miRNAs are abundant in the liver and play very important roles in liver functions (Chen 2009), and modulation of miRNA expression profiles is closely linked to the biological and clinical behavior of human intrahepatic CC (Chen et al. 2009).

The aim of this study was to test the hypothesis that furan can modulate the expression of genes relevant to tumor induction, possibly through effects on DNA methylation or miRNA expression in rat liver. Importantly, we treated rats with 0.1 mg/kg bw furan, a dose closer to the estimated highest level of human exposure (3.5 μg/kg bw/day) (European Food Safety Authority 2004), and with 2 mg/kg bw furan, the lowest dose associated with rat carcinogenicity (Maronpot et al. 1991). We used furan-induced CC and paired nontumor tissues as reference samples for DNA methylation assays.

## Materials and Methods

### Chemicals

We obtained furan (CAS no. 110-00-9, ≥ 99% pure) from Sigma-Aldrich (Munich, Germany), NovaTaq Hot Start DNA Polymerase (catalog no. 71091) from Merck (Darmstadt, Germany), and all other chemicals and enzymes from New England Biolabs (Ipswich, MA, USA) or Sigma-Aldrich, if not mentioned otherwise.

### Animals

Male F344/N rats at 6–7 weeks old were purchased from Harlan-Winkelmann (Borchen, Germany). Animals were treated humanely and with regard for alleviation of suffering. All procedures involving animals were performed according to national animal welfare regulations after authorization by the local authorities (Regierung von Unterfranken). All animals were given free access to pelleted standard rat maintenance diet and tap water, and were housed in groups of five in Makrolon cages (TECNIPLAST, Hohenpeißenberg, Germany) at 21 ± 2°C and a 12/12 hr day/night cycle. Rats were allowed to acclimatize for 5–7 days before furan treatment. Furan was prepared in corn oil vehicle immediately before use and was administered orally via gavage at doses of 0, 0.1, and 2 mg/kg bw for 5 days, 28 days, and 28 days plus a 14-day recovery period, for a total of nine treatment groups; animals were treated 5 days/week. Sections of left lobes of the liver, including subcapsular proliferative regions [which have previously been shown to be susceptible to furan-induced tumor formation (Mally et al. 2010; Maronpot et al. 1991)] were removed, frozen in liquid nitrogen, and stored at −80°C.

### Genomic DNA and RNA purification

Samples of frozen rat liver (30 mg; *n* = 5) were processed using Precellys kit 03961CK14 (Bertin Technologies, Montigny-le-Bretonneux, France) containing 0.6 mL RLT buffer (Qiagen, Hilden, Germany) with 6 μL β-mercaptoethanol. After loading on the Precellys 24 system (Bertin Technologies), samples were homogenized and centrifuged two times at 2,400 rpm for 10 sec. Genomic DNA and RNA were purified at the same time using the AllPrep DNA/RNA Mini Kit (Qiagen) according to the manufacturer’s instructions. Genomic DNA contamination was removed from purified RNA samples using the Turbo DNA-free kit (Applied Biosystems, Foster City, CA, USA). The concentrations of DNA and RNA were measured by ultraviolet (UV) absorbance using a NanoDrop 1000 Spectrophotometer (Thermo Scientific from Fisher Scientific, Loughborough, UK). The purity of DNA and RNA were determined by UV scanning between 200 and 300 nm and by the 260:280 nm ratio. For DNA and RNA, the ratios were 1.8–1.9 and 1.9–2.0, respectively. RNA quality was assessed by RNA gel electrophoresis.

### mRNA polymerase chain reaction (PCR) array

One microgram of purified RNA was used to synthesize complementary DNA using the RT^2^ Profiler PCR Array kit (PARN-012, -020, and -029; SABiosciences, Frederick, MD, USA). We performed real-time PCR using an ABI7000 PCR system and PowerSYBR reagents (Applied Biosystems) following the standard two-step cycling and dissociation program. Results from three different rat liver samples in each group were analyzed using the Excel-based data analysis template provided by SABioscience. Relative gene expression was calculated using the comparative threshold cycle (Ct) method (2^−ΔΔCt^).

### miRNA PCR array

We extracted total RNA containing miRNA from three different rat liver samples in each group using a *mir*Vana miRNA isolation kit (Applied Biosystems). Purified RNA (2 μg) was reverse transcribed into complementary DNA using a QuantiMir Kit (RA680A-1; System Biosciences, Mountain View, CA, USA). Real-time PCR was performed on a 384-well plate using an ABI7900 PCR system (Applied Biosystems) and a QuantiMir kit (System Biosciences) and PowerSYBR reagents (Applied Biosystems) following standard thermocycling conditions. We calculated relative miRNA expression (Ct) using the 2^−ΔΔCt^ method. We used miRGen software (Megraw et al. 2007) and MicroCosm Targets software (version 5; European Molecular Biology Laboratory, European Bioinformatics Institute 2010) to predict miRNA targets.

### Methylation-specific PCR (MSP) and combined bisulfite restriction analysis (COBRA)

To generate positive methylated DNA controls, we incubated rat genomic DNA with 4 U *M.SssI* (CpG methylase) in the presence of *S*-adenosylmethionine. Bisulfite conversion of genomic DNA was performed using an EZ DNA Methylation-Gold Kit (Zymo Research, Orange, CA, USA) following the manufacturer’s protocol. Genomic DNAs (~ 400 ng) from five different samples in each group were used for bisulfite treatment. Elution volume was 40 μL, and 2 μL of the eluted DNA was used as a PCR template. PCR reactions were performed on a Mastercycler (Eppendorf, Hamburg, Germany); primers are listed in Supplemental Material, Table 1 (doi:10.1289/ehp.1002153). For MSP, the PCR products were directly run on a 2% agarose gel. For COBRA, the PCR products were digested by the corresponding restriction enzymes and then run on a 2% agarose gel.

### Methylated DNA immunoprecipitation (MeDIP) and microarray analysis

We used this method as an “open” unbiased system to detect methylation changes. Methylated DNA was immunoprecipitated using the MagMeDIP kit (mc-magme-048; Diagenode, Liège, Belgium). Briefly, genomic DNA purified from rat liver was sheared to 200–1,000 bp at 30% power for ten 15-sec pulses using VCX130 (Sonics, Newtown, CT, USA) and then incubated with anti–5-methylcytosine antibody at 4°C overnight. After washing four times, the methylated DNA-enriched genomic DNA faction was eluted in provided elution buffer. For microarray analysis, the immunoprecipitated DNA and input DNA were amplified with a whole genome amplification kit (WGA2-50RXN; Sigma-Aldrich) and then were sent to NimbleGen’s service laboratory to perform the array experiments using Rat CpG Island Promoter microarray (C7120-00-01; Roche NimbleGen, Madison, WI, USA). Data were extracted from scanned images using NimbleScan 2.0 extraction software (Roche NimbleGen) and were entered into GeneSpring GX software (version 7.3; Agilent Technologies, Santa Clara, CA, USA) for analysis.

### Liquid chromatography/tandem mass spectrometry (LC-MS/MS)

Global DNA methylation was detected in at least five different rat liver samples in each group. RNA contamination of isolated DNA was removed by treating with 100 μg/mL RNase A and 2,000 U/mL RNase T in a final volume of 100 μL at 37°C for 2 hr; DNA was then purified by phenol/chloroform extraction followed by ethanol precipitation. Genomic DNA (5 μg) was then denatured at 100°C for 3 min and chilled on ice. After treating with 5 U nuclease P1 (Calbiochem, Darmstadt, Germany) for 1 hr at 37°C, 1 U alkaline phosphatase (Sigma-Aldrich, St. Louis, MO, USA) was added to the sample and incubated for 30 min at 37°C. The hydrolyzed DNA was transferred onto a cutoff filter (Millipore, Billerica, MA, USA) and centrifuged for 20 min at 4°C and 14,000 rpm. DNA hydrolysate (5 μL) was diluted with 495 μL double-distilled H_2_O in a vial. LC-MS/MS analysis was performed using an Agilent 1100 series LC coupled to an API 3000 triple quadrupole mass spectrometer equipped with a turbo ion spray source (Applied Biosystems). Separation was carried out on a Reprosil Pur ODS 3 column (150 × 2 mm, 5 μm) by gradient elution with 0.1% formic acid (solvent A) and methanol (solvent B) using the following conditions: 90% A and 10% B (starting conditions) followed by an increase to 40% in 3 min and a linear increase to 100% B in 2.5 min, at a flow rate of 0.3 mL/min. Analytes were detected in the positive ion mode at a vaporizer temperature of 400°C. Data acquisition was performed by multiple reaction monitoring of mass transitions *m*/*z* 268.2 to 152.1 for 2-deoxyguanosine, and *m*/*z* 242.17 to 126.0 and *m*/*z* 242.17 to 108.95 for 5-methyldeoxycytidine. Quantitation of 2-deoxyguanosine and 5-methyldeoxycytidine was performed using external standards.

### Statistical analyses

We used the Student *t*-test to analyze PCR arrays for both mRNA and miRNA expression profiles; only expression changes that were statistically significant and at least two times higher or lower than expression in controls were considered. mRNAs and miRNAs with average Ct values > 32 in both control and treated groups were not investigated further. Global DNA methylation levels were compared using one-way analysis of variance followed by Dunnett’s and Tukey’s multiple comparison test. A *p* value < 0.05 was considered statistically significant.

## Results

### mRNA expression profiles of genes related to apoptosis, cell cycle, and DNA damage

Each PCR array contained 84 genes related to apoptosis, cell cycle, and DNA damage. Three genes (*casp3*, *Bcl2*, and *Trp63*) are important to both apoptosis and cell cycle and were included in both arrays. Twelve other genes were present in both cell cycle and DNA damage arrays. Moreover, *Tp53* and *Gadd45a* existed in all three arrays. Thus, in total, we examined 233 genes in this study using PCR arrays [see Supplemental Material (doi:10.1289/ehp.1002153)]. The expression of 18 genes, including *Cdnk2a*, was barely detectable in both control and treated samples (with an average Ct value > 32; data not shown); expression of these genes was not considered further.

**Figure 1:**
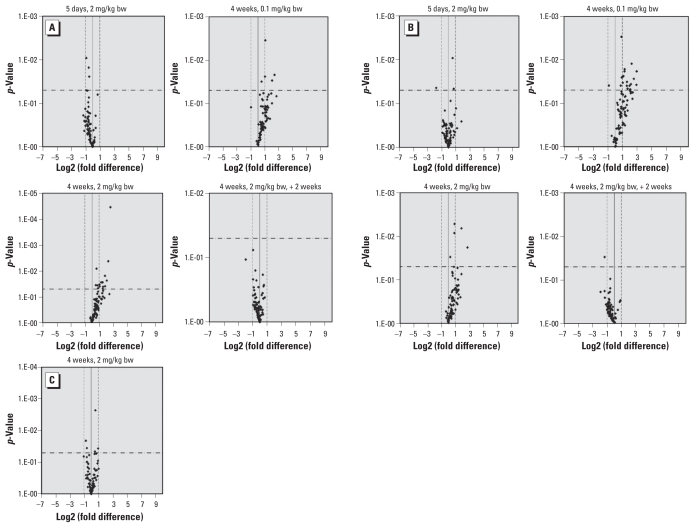
Volcano plots of relative changes in the expression of genes related to apoptosis (*A*), cell cycle (*B*), and DNA damage (*C*) in liver from rats treated with furan at 0.1 or 2 mg/kg/bw for 5 days, 4 weeks, or 4 weeks plus a 2-week recovery period (*n* = 3 animals per treatment group). The solid vertical line indicates a relative change in gene expression of 1 (i.e., no difference in expression relative to untreated controls); dashed vertical lines indicate expression levels 2 times higher or lower than controls; and the horizontal dash-dot line indicates the threshold for *p* < 0.05. For a complete list of the genes evaluated in each group, see Supplemental Material (doi:10.1289/ehp.1002153).

As shown in Figure 1A and B and in Supplemental Material, Table 2 (doi:10.1289/ehp.1002153), at doses 0.1 and 2 mg/kg bw, no genes were significantly expressed at levels two times higher or two times lower than those in controls after 5 days of furan treatment, with the exception of *Npm2*, a cell-cycle–related gene that showed a slight down-regulation (relative expression, −3.39; *p* = 0.044). In contrast a number of apoptosis-related and cell-cycle–related genes were overexpressed after 4 weeks of furan treatment. Altered expression of apoptosis-related genes was more common after treatment at 2 mg/kg bw than 0.1 mg/kg bw (Figure 1A; see also Supplemental Material, Table 2). However, 14 cell-cycle–related genes were statistically significantly up-regulated after furan treatment at 0.1 mg/kg bw, compared with only two genes after 2 mg/kg bw furan (Figure 1B; see also Supplemental Material, Table 2). All up-regulated genes returned to control levels of expression 2 weeks after withdrawal of furan treatment. For DNA-damage–related genes, we found no change in mRNA expression in rat liver treated with furan at 2 mg/kg bw (Figure 1C). We did not perform DNA damage PCR arrays in samples treated with 0.1 mg/kg bw furan, but *Brca2* (relative expression, 2.46; *p* < 0.05) and *Chek1* (relative expression, 2.05; *p* < 0.05) genes, related to both cell cycle and DNA damage, did show slight up-regulation (Supplemental Material, Table 2).

### miRNA expression profiles

Of the 349 miRNA we examined, 64 had average Ct values > 32 in both control and treated samples [see Supplemental Material (doi:10.1289/ehp.1002153)]; we did not further consider expression of these genes. As shown in Table 1, expression of 13 miRNAs was > 2 times higher or lower than in controls (*p* < 0.05), including only two miRNAs (rno-let-7a and rno-miR-28) that were up-regulated. Using miRGen and MicroCosm, we found that *Ccna2* and *Bcl10*, two genes that were up-regulated in our mRNA arrays, were the predicted targets of the down-regulated rno-miR-489 and rno-let-7e*, respectively. *E2f1*, which was significantly up-regulated after treatment with 0.1 mg/kg bw furan, was the predicted target of down- regulated rno-miR-296.

### Analysis of DNA methylation of selected genes

**Figure 2:**
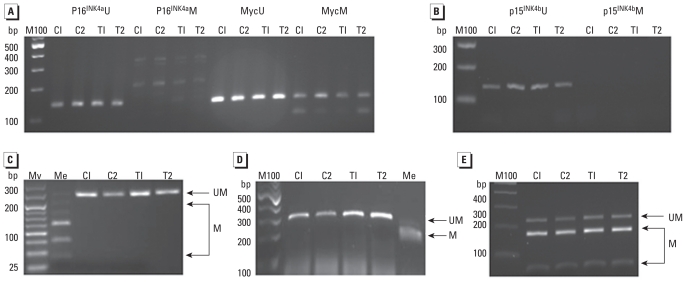
DNA methylation status of *p16^INK4a^*, *p15^INK4b^*, *Myc*, *Bid3*, and *Sfn* promoter region in liver samples from control rats (C1, C2) or rats treated with furan (2 mg/kg bw for 4 weeks; T1, T2) (*n* = 5 rats). MSP profiles of *p16^INK4a^* and *Myc* promoter region (*A*) and *p15^INK4b^* promoter (*B*). COBRA results of *c-myc* gene digested by *TaqI* 241 bp (40/77/125) (*C*), *Bid3* gene digested by *BstUI* 310 bp (210/68/32) (*D*), and *Sfn* gene digested by *TaqI* 229 bp (56/173) (*E*). Abbreviations: bp, base pairs; C, cleaved PCR products; M100, 100-bp DNA ladder (New England Biolabs); Me, methylated rat genomic DNA treated with methyltransferase (M.SssI); MV, HyperLadder V (Bioline, Luckenwalde, Germany); UM, uncleaved PCR products.

To determine if there was any methylation change in furan-treated samples at the 2 mg/kg bw dose, we first examined the methylation status of five genes relevant to carcinogenesis: *p16^INK4a^*, *p15^INK4b^* (*Cdkn2b*), *Bid3* (BH3 interacting domain), *Myc* (myelocytomatosis oncogene), and *Sfn* (stratifin, also known as 14-3-3 sigma). Two of these genes (*p15^INK4b^* and *Bid3*) were up-regulated in furan-treated rat liver samples based on mRNA PCR arrays, consistent with the hypothesis that methylation may, in part, contribute to the mechanism of action. *Myc* is an oncogene, whereas *Sfn*, *p16^INK4a^*, and *p15^INK4b^* are tumor suppressor genes. MSP results shown in Figure 2A and B demonstrate that both *p16^INK4a^* and *p15^INK4b^* promoter regions were unmethylated in all control and treated samples, and only a very small proportion of *Myc* promoter region was methylated. COBRA results (Figure 2C) further proved the low methylation status of *Myc* promoter in both control and treated rat liver. Lack of methylation of *Bid3* and methylation of *Sfn* in all control and treated samples were also demonstrated in COBRA results (Figure 2D). Overall, we found no furan-induced DNA methylation changes in the selected genes.

### MeDIP and DNA methylation microarray results

To screen for alternative genes with potential methylation alteration, we conducted MeDIP and microarray experiments. We focused on the 2 mg/kg bw furan treatment group because this dose can induce CC after 2 years of treatment (Maronpot et al. 1991). R. Maronpot kindly provided a furan-induced CC sample from a female Sprague-Dawley rat treated with 2 mg/kg bw furan for 500 days that we used as a reference sample and in which we found modulation of methylation.

After immunoprecipitation, we examined the enrichment of four single-copy genes as well as the positive and negative controls provided in the Diagenode MeDIP kit to confirm the enrichment of methylated DNA by anti–5-methylcytosine antibody. The hypomethylation status of *Myc* gene promoter region was independently shown by MSP and COBRA (Figure 2A,C), whereas the 5′-upstream region of the *H19* gene has been found to be highly methylated in the adult rat by bisulfite sequencing (Manoharan et al. 2004). Thus, *Myc* and *H19* could be employed as negative and positive controls, respectively. We also investigated the enrichment rate of LINE-1 (long interspersed nucleotide element type 1) and ID (identifier) elements because repetitive elements represent a large part of the genomes and can reflect global methylation level to some extent. ID elements are members of a family of SINEs (short interspersed nucleotide elements) in rodents. We speculate that cytosines lie within the CpG islands of the transposons, including LINE and SINE. Only the *H19* gene and the positive internal control were enriched in immunoprecipitated DNA [see Supplemental Material, Figure 1 (doi:10.1289/ehp.1002153)]. LINE-1 showed little enrichment, which is consistent with a previous report that LINE-1 was hypomethylated in rat liver (Asada et al. 2006). Although the methylation level of one CpG of ID elements has been reported at > 60% (Kim et al. 2007), considering the high mutation level and the possible low methylation status of other CpG sites, it is not surprising that few ID elements were enriched in immunoprecipitated DNA.

We found no evident methylation change in samples treated with furan for 4 weeks compared with control samples by the microarray method. However, when validating the assay, we noted methylation changes in promoter regions of the CC sample relative to paired normal liver samples (data not shown).

### Global DNA methylation

**Figure 3:**
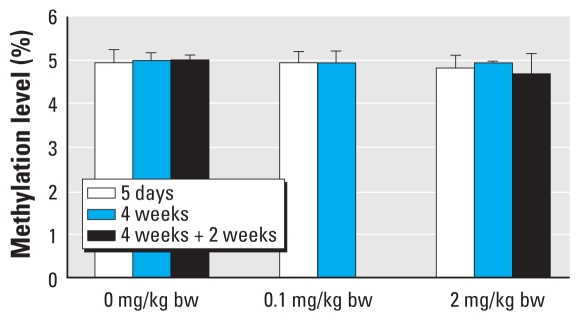
Global methylation level in liver samples from furan-treated rats (*n* = 5 animals per treatment group).

We observed no significant global methylation change in either furan treatment group (Figure 3). However, CC samples showed slight but significant hypomethylation levels compared with nontumor samples (*p* < 0.01; Figure 3). Global methylation in mammals usually ranges from 3% to 5% (Bombail et al. 2004; Özden et al. 2008), in agreement with our findings.

## Discussion

We assessed gene expression changes and epigenetic parameters relevant to carcinogenesis at furan doses lower than the minimum dose that has been shown to cause tumors, and closer to estimated exposure levels in humans. Although treatment with 2 mg/kg bw furan for 5 days did not appear to influence gene expression, we observed statistically significant expression changes in the expression of a number of apoptosis-related and cell-cycle–related genes after 4 weeks of treatment with 0.1 mg/kg bw furan. We also found that changes in gene expression following 4 weeks of treatment with 2 mg/kg bw furan were reversed 2 weeks after furan treatment was discontinued, which suggests that sustained exposure to furan is required to elicit effects at relatively low doses. Consistent with this, Hamadeh et al. (2004) reported a significant gene expression difference between 2-week exposure and 3- or 7-day exposure in rats treated with 4 mg/kg furan.

Although we found no DNA-damage–related gene expression changes in samples from animals treated with 2-mg/kg bw furan, *Brca2* and *Chek1*—two cell-cycle–related genes that respond to DNA double-strand breaks (DSBs)—showed a slight overexpression in samples from animals treated with 0.1 mg/kg bw furan. *cis*-2-Butene-1,4-dial, the major reactive metabolite of furan, has been shown to form covalent adducts with nucleosides *in vitro* (Byrns et al. 2006), and DSBs have been found in mitogen-stimulated splenocytes of furan-treated mice (Leopardi et al. 2010). High-dose (30 mg/kg bw) and long-term (3 months) treatment with furan has been found to induce oxidative stress and associated DNA damage (Hickling et al. 2010). However, in the present study, which was shorter term and used a lower dose, we found no change in oxidative DNA-damage–related gene expression, nor did we find a gene expression profile characteristic of a range of genotoxic carcinogens, such as described by Ellinger-Ziegelbauer et al. (2005). In another study (Mally et al. 2010), we observed only a reversible enhanced proliferation in the subcapsular region of the left liver lobe after treatment with 2 mg/kg bw furan for 4 weeks, with little or no DNA oxidation. Considering that oxidative DNA damage is related to promoting activity more than initiating activity (Ellinger-Ziegelbauer et al. 2005; Umemura et al. 1999), the oxidative DNA damage may be a secondary mechanism associated with a persistent inflammatory response (Hickling et al. 2010).

Recent studies indicate that miRNAs are involved in regulation of cell proliferation and apoptosis (Ambros 2004). Moreover, miRNA expression profiles have been closely associated with the biological and clinical behavior of human intrahepatic CC (Chen et al. 2009). Thus, altered miRNA expression might contribute to the deregulation of certain cell-cycle–related and apoptosis-related genes. Here we found that 13 of the 349 miRNAs examined were aberrantly expressed in samples from the 2-mg/kg–treated animals, and two down-regulated miRNAs (rno-let-7e* and rno-miR-489) were consistent with the up- regulation of mRNA expression of their predicted target genes, *Bcl10* and *Ccna2*. The *let-7* family of miRNAs, which is functionally conserved from worms to humans, is important to normal development and differentiation and has been reported to be deregulated in various cancers (reviewed by Boyerinas et al. 2010). For example, let-7e* was down-regulated in malignant mesothelioma (Guled et al. 2009), and *let-7a* was up-regulated in lung, lymphoma, and ovarian cancers (Boyerinas et al. 2010). *Caspase-3*, *Dicer*, and *Myc* have been confirmed to be *let-7a* targets (Boyerinas et al. 2010). In the present study, rno-miR-296 showed the greatest change in expression (~ 8-fold down-regulation). Human *miR-296* has been reported to be down-regulated in breast cancer and parathyroid cancer (Barh et al. 2008; Corbetta et al. 2010). Furthermore, inhibition of miR-296 in Hela cells has been reported to decrease cell growth and increase the level of apoptosis (Cheng et al. 2005).

Changes in DNA methylation constitute a mechanism for altering gene expression and are important nongenotoxic mechanisms contributing to cancer. In the present study, we investigated the methylation status of five genes (*p16^INK4a^*, *p15^INK4b^*, *Bid3*, *Myc*, and *Sfn*) in samples from rats treated with furan at 2 mg/kg bw. Promoter methylation changes of *p16^INK4a^*, *p15^INK4b^*, and *Sfn* have been reported in CC (Lee et al. 2002; Yang et al. 2005), and hypomethylation-induced *Myc* overexpression has been found in various types of tumors (Del Senno et al. 1989; Tao et al. 2002; Tsujiuchi et al. 1999). However, in the present study, we found no DNA methylation change in the five genes evaluated by MSP and/or COBRA. Furthermore, we found no methylation changes using the “open” unbiased method of MeDIP and microarray assay using NimbleGen’s Rat CpG Island Promoter microarray, which covers 15,809 islands and 14,490 promoters. In addition, we did not observe evidence of a global methylation change based on LC-MS/MS. Taken together, these results suggest that the reversible gene expression changes we observed were not caused by methylation changes. The findings contrast with the observation of irreversible gene expression changes, a DNA damage response, and altered methylation of at least one gene in liver samples from rats treated with a much higher furan dose of 30 mg/kg bw for 3 months (Hickling et al. 2010; Chen T, Williams T, Mally A, Hamberger C, Hickling K, Chipman JK, unpublished data).

## Conclusion

Short-term treatment with furan at a dose approaching the maximum estimated human dietary intake resulted in reversible changes in the expression of genes that control the cell cycle and cell death but did not appear to influence the expression of genes involved in responses to DNA damage. These changes did not appear to result from changes in DNA methylation, although modulation of miRNA may have a minor role in these responses. Bearing in mind the demonstrated differential profile of gene expression of genotoxic and nongenotoxic carcinogens in rodent liver (Ellinger-Ziegelbauer et al. 2005), the cell cycle and apoptotic responses we observed may provide a nongenotoxic influence on carcinogenesis at furan doses lower than the currently identified lowest carcinogenic dose. Further related studies on low doses of furan are under way by the NTP (2008).

## Figures and Tables

**Table 1:** miRNAs with > 3-fold significant expression change in liver of rats treated with furan at 2 mg/kg bw for 4 weeks.

miRNA	*p*-Value	Change relative to controls
rno-let-7a	0.0061	2.63
rno-let-7e*	0.0459	−2.05
rno-miR-28	0.0132	3.08
rno-miR-129*	0.0159	−2.39
rno-miR-187	0.0273	−2.23
rno-miR-207	0.0076	−4.09
rno-miR-298	0.0311	−2.06
rno-miR-296	0.0448	−7.93
rno-miR-376b-3p	0.0438	−2.44
rno-miR-488	0.0220	−2.07
rno-miR-489	0.0325	−3.55
rno-miR-493	0.0063	−5.64
rno-miR-598–5p	0.0173	−2.47
